# Ovariectomy Increases the Participation of Hyperpolarizing Mechanisms in the Relaxation of Rat Aorta

**DOI:** 10.1371/journal.pone.0073474

**Published:** 2013-09-13

**Authors:** Ana Sagredo, Lara del Campo, Aina Martorell, Rocío Navarro, María C. Martín, Javier Blanco-Rivero, Mercedes Ferrer

**Affiliations:** 1 Departamento de Fisiología, Facultad de Medicina, Universidad Autónoma de Madrid, Madrid, Spain; 2 Instituto de Investigaciones Sanitarias IdiPAZ, Madrid, Spain; Universität Regensburg, Germany

## Abstract

This study examines the downstream NO release pathway and the contribution of different vasodilator mediators in the acetylcholine-induced response in rat aorta 5-months after the loss of ovarian function. Aortic segments from ovariectomized and control female Sprague-Dawley rats were used to measure: the levels of superoxide anion, the superoxide dismutases (SODs) activity, the cGMP formation, the cGMP-dependent protein kinase (PKG) activity and the involvement of NO, cGMP, hydrogen peroxide and hyperpolarizing mechanisms in the ACh-induced relaxation. The results showed that ovariectomy did not alter ACh-induced relaxation; incubation with L-NAME, a NO synthase inhibitor, decreased the ACh-induced response to a lesser extent in aorta from ovariectomized than from control rats, while ODQ, a guanylate cyclase inhibitor, decreased that response to a similar extent; the blockade of hyperpolarizing mechanisms, by precontracting arteries with KCl, decreased the ACh-induced response to a greater extent in aortas from ovariectomized than those from control rats; catalase, that decomposes hydrogen peroxide, decreased the ACh-induced response only in aorta from ovariectomized rats. In addition, ovariectomy increased superoxide anion levels and SODs activity, decreased cGMP formation and increased PKG activity. Despite the increased superoxide anion and decreased cGMP in aorta from ovariectomized rats, ACh-induced relaxation is maintained by the existence of hyperpolarizing mechanisms in which hydrogen peroxide participates. The greater contribution of hydrogen peroxide in ACh-induced relaxation is due to increased SOD activity, in an attempt to compensate for increased superoxide anion formation. Increased PKG activity could represent a redundant mechanism to ensure vasodilator function in the aorta of ovariectomized rats.

## Introduction

Vascular endothelium plays a critical role regulating vascular tone by releasing relaxing and contracting factors [1]; among these factors, nitric oxide (NO) is of singular relevance [2]. One of the major downstream events occurring after NO release is an increase in cGMP formation through soluble guanylate cyclase stimulation, subsequent activation of cGMP-dependent protein kinase (PKG) [3] which reduces the intracellular calcium concentration through a wide spectrum of PKG substrates, leading to vasodilation [4,5]. Concerning NO, it is important to take into account that vascular functionality of endothelial NO depends on its bioavailability, which is determined by the rate of NO production and by the rate it is scavenged by superoxide anions. Therefore, the elimination of superoxide anions from the vessel wall by superoxide dismutases (SODs) is an essential function [6]. It is well understood that alterations in any step of the NO pathway determines its effect on the vascular tone.

In addition to the activation of the cGMP-PKG pathway, NO may stimulate vascular hyperpolarization [7]. Hyperpolarizing factors/mechanisms are also important regulators of the membrane potential and hence of vessel tone [2]. The release of an endothelium derived hyperpolarizing factor (EDHF) has been proposed and, although the nature of EDHF remains to be defined, different studies have suggested that hyperpolarization may result from endothelial release of different substances [8-10]. Thus, hyperpolarization induced by NO [7], cGMP [11], superoxide anion [12] and other reactive oxygen species [13] has been reported.

Clinical studies have shown the important role of endogenous sex hormones in the control of cardiovascular function in men [14] and women [15-17]. Therefore, our work has been focused on trying to elucidate the alterations in different cell signalling pathways that take place when the ovarian function is lost [18,19] instead of studying direct vascular effects of specific sex hormones. We have previously demonstrated that the loss of ovarian function increases the production of prostanoids derived from cyclooxygenase-2 (COX-2), while it does not modify the release of nitric oxide (NO) [19]. Additionally, we already suggested that complementary cell signalling pathways, such as the NO-cGMP pathway, could be working simultaneously to maintain the vasodilatory effect of acetylcholine (ACh).

Although modulatory actions of several female sex hormones on NO, cGMP formation [20-22] and hyperpolarizing mechanisms [12] have been reported, there is no information about changes induced by the loss of ovarian function, especially for prolonged periods after ovariectomy. In view of these data, the objective was to analyze how the loss of ovarian function influences the balance between formation/degradation of superoxide anion and the pathway downstream NO release, specifically cGMP formation and PKG activity. The contribution of NO, cGMP and hyperpolarizing mechanisms in the vasodilator response induced by acetylcholine (ACh) was also studied.

## Materials and Methods

### Animal housing and protocols

Female Sprague-Dawley rats (6 months old) were divided into two groups: control (in oestrus phase) and ovariectomized rats. All animals were housed in the Animal Facility of the Universidad Autónoma de Madrid (Registration number EX-021U) according to directives 609/86 CEE and R.D. 233/88 of the Ministerio de Agricultura, Pesca y Alimentación of Spain. Bilateral ovariectomy was induced at 4 weeks of age under anesthesia by isoflurane inhalation. The adequacy of anesthesia was tested by observing regular respiratory rhythm and absence of retraction reflex in hind-legs after mechanical stimulation. Rats were treated with 0.30 mg/Kg SC meloxicam (Metacam 5mg/ml; Boehringer-Ingelheim) immediately after surgery and with 50 mg/Kg ibuprofen, orally administered for 4 days. Systolic blood pressure was indirectly measured in awake animals by the tail-cuff method [19] using a Letica, Digital Pressure Meter LE5000 (Barcelona, Spain). The body weight was obtained on the day of the experiment, before animals were sacrificed. Since the plasma level of sex hormones varies along the day, the time at which the oestrus stage was determined (by microscopic evaluation of a vaginal smear taken before the animals were sacrificed) was also the same. Rats were sacrificed 5 months after surgery by CO_2_ inhalation; the thoracic aorta was carefully dissected out, cleaned of connective tissue, cut into 4 mm long segments and placed in Krebs-Henseleit solution (KHS) at 4 °C containing, in mmol/L: NaCl 115, CaCl_2_ 2.5, KCl 4.6, KH_2_PO_4_ 1.2, MgSO_4_ 1.2, NaHCO_3_ 25, glucose 11.1, Na_2_ EDTA 0.03. The uterus was trimmed and weighed after excess of connective tissue was eliminated, to confirm the effectiveness of ovariectomy. The investigation conforms to the *Guide for the Care and Use of Laboratory Animals* published by the USA National Institutes of Health (NIH publication No. 85.23 revised 1985). This study was also approved by the Ethical Committee of the Universidad Autónoma of Madrid.

### Detection of superoxide anion

Hydroethidine (HE), an oxidative fluorescent dye, was used to evaluate superoxide anion levels *in situ*, as previously described [23]. Briefly, aortic rings from control or ovariectomized rats were cryoprotected with 30% (w/v) sucrose in PBS, frozen and embedded in optimum cutting temperature compound, OCT Tissue Tek, and 20 µm cryostat sections were obtained. Aortic sections from control and ovariectomized rats were incubated with HE (5 µmol/L) in a light-protected, humidified chamber at 37 °C for 30 min and simultaneously processed. Hydroethidine, that fluorescens red when oxidized to EtBr was imaged with a LEICA (TCS ST2 DM IRE2) laser scanning confocal microscope (excitation 488 nm, emission 610 nm). Laser and image settings were fixed for the acquisition of images from the two groups. The photomicrographs show the intensity and location of the oxidized HE, which reflects the levels of superoxide anion, so that comparison between groups can be made. To analyse fluorescence intensity the ImageJ Analysis Software (National Institutes of Health) was used.

Superoxide anion levels were also measured using lucigenin chemiluminescence, as previously described [23]. Briefly, aortic segments were rinsed in KHS for 30 min, equilibrated for 30 min in HEPES buffer at 37 °C, transferred to test tubes that contained 1 ml HEPES buffer (pH 7.4) containing lucigenin (5 µmol/L) and then kept at 37 °C during 30 minutes. The luminometer was set to report arbitrary units of emitted light; repeated measurements were collected during 5 min at 10 s intervals and averaged. 4,5-dihydroxy-1,3-benzene-disulphonic acid “Tiron” (10 mmol/L), a cell permeant, non-enzymatic scavenger of the superoxide anion, was added to quench the superoxide anion-dependent chemiluminescence. Also blank measures were collected in the same way without aortic segments to substract background emission. Some assays were performed in the presence of SOD (13 units/ml) to ensure the specificity of the method.

### Superoxide dismutase activity

Frozen samples of aortic segments were homogenized in a buffer composed (in mmol/L) of HEPES 20, EGTA 1, mannitol 210 and sucrose 70. After centrifugation at 15000 g for 5 min, 5-10 µl of supernatants were used to the assay. The enzyme activity was measured by a SOD-assay kit (Cayman Chemical) following the manufacturer’s instructions. The SOD activity was expressed as units/ml μg protein.

### Determination of cGMP

Aortic segments were subjected to a resting tension of 1 g. After an equilibration period of 60 min, segments were contracted with 0.1 µmol/L noradrenaline (NA) during 3 min (considered the basal level), and then some segments were incubated with 10 µmol/L ACh for 15 s. Segments were immediately frozen in liquid nitrogen and stored at -70 °C. Levels of cGMP were determined using the cGMP Enzyme Immunoassay Kit from Assay Designs. For this assay, the frozen arteries were homogenized in 0.1 mol/L HCl and centrifuged at 600 g for 10 min at 4 °C. The non-soluble fraction was used to measure protein content with a DC protein assay kit (Bio-Rad). The supernatant was then collected and used for the assay. cGMP levels were measured following the manufacturer’s protocol. Results were expressed as pmol cGMP/mg protein.

### Activity of PKG

Aortic segments from control and ovariectomized rats were frozen in liquid nitrogen and stored at -70 °C. PKG activity was measured using the comercial kit CycLex^®^ Cyclic GMP dependent protein Kinase Assay Kit. The frozen arteries were homogenized in samples prepared according to the manufacturer’s protocol. Non-soluble fractions were used to measure protein content with a DC protein assay kit (Bio-Rad). The supernatant was then collected and used for the assay, following the manufacturer’s instructions. Results were expressed as arbitrary units/mg protein.

### Vascular reactivity

The method used for isometric tension recording has been described in full elsewhere [24]. Briefly, two parallel stainless steel pins were introduced through the lumen of the vascular segment: one was fixed to the bath wall, and the other connected to a force transducer (Grass FTO3C; Quincy, Mass., USA); this was connected in turn to a model 7D Grass polygraph. Segments were suspended in an organ bath containing 5 ml of KHS at 37 °C continuously bubbled with a 95% O_2_-5% CO_2_ mixture (pH 7.4). The segments were subjected to a tension of 1 g which was readjusted every 15 min during a 90 min equilibration period before drug administration. After this, the vessels were exposed to KCl (75 mmol/L) to check their functional integrity. After a washout period, the presence of vascular endothelium was tested by the ability of ACh (10 µmol/L) to relax segments precontracted with NA (0.1 µmol/L).

After this, the segments were rinsed several times with KHS over 1 h period, and then cumulative concentration-response curves to ACh (0.1 nmol/L -10 µmol/L), to the NO donor sodium nitroprusside (SNP, 0.1 nmol/L -10 µmol/L) or to the 8Br-cGMP (0.1 µmol/L -10 mmol/L) were obtained in NA-precontracted segments (NA, 0.1 µmol/L). To analyse the participation of NO or cGMP on the response induced by ACh, the NO synthase inhibitor L-NAME (0.1 mmol/L) [19] or the guanylate cyclase inhibitor ODQ (10 µmol/L) was added 30 min before the concentration-response curves were performed.

To study the possible contribution of hyperpolarizing mechanisms to the vasodilator response to ACh or to SNP, some arterial segments were precontracted with 30 mmol/L of KCl before performing the concentration-response curves to ACh or SNP.

To analyze the involvement of endogenous hydrogen peroxide in the relaxation induced by ACh or SNP, arteries were incubated with catalase (1000 U/ml) 30 min before the NA- or KCl-induced precontraction and subsequent concentration-response curves to ACh or SNP were performed. μStatistical analysis

Results are given as mean ± S.E.M. (Standard Error of the Mean). The responses elicited by KCl and NA were expressed in mg. The relaxations induced by ACh, SNP or 8Br-cGMP were expressed as a percentage of the initial contraction elicited by NA or KCl. Statistical analysis was done by comparing the curve obtained among groups and in the presence of L-NAME or ODQ with the control curve by means of two-way analysis of variance (ANOVA). The maximum response (E_max_ value) and the logarithm of the ACh concentration producing 50% of maximum response (log EC_50_) were calculated by a non-linear regression analysis of each individual concentration-response curve using Graph Pad Prism Software (San Diego, CA, USA) and the statistical analysis was done used unpaired Student’s *t*-test. To compare the effect of drugs on ACh-induced responses in aortic segments from control and ovariectomized rats, certain results are expressed as differences in the area under the concentration-response curves (dAUC) between control and experimental conditions. The differences, expressed as a percentage of the control AUC, were analysed using the Student’s *t*-test. For the experiments on cGMP formation, PKG and SOD activity, the statistical analysis Student’s *t*-test was also performed. A *p* value of less than 0.05 was considered significant.

### Drugs

Drugs used were: L-NA hydrochloride, ACh chloride, L-NAME hydrochloride, ODQ, catalase, sodium nitroprusside and 8Br-cGMP (Sigma-Aldrich). Stock solutions (10 mmol/L) of drugs were prepared in distilled water, except for NA which was dissolved in NaCl (0.9%)-ascorbic acid (0.01% w/v) solution. These solutions were kept at -20 °C and appropriate dilutions were made in KHS or HEPES-buffer on the day of the experiment.

## Results

### Detection of superoxide anion

**Figure 1:**
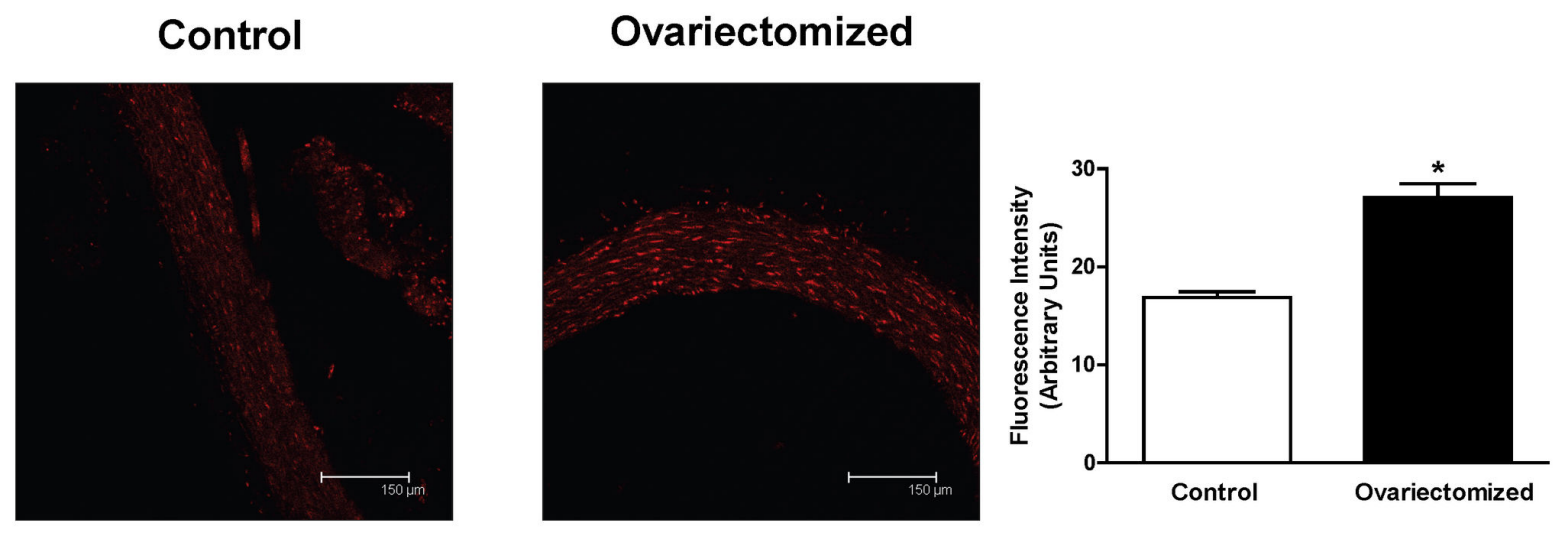
Effect of superoxide anion formation. Confocal micrographs showing in situ detection of superoxide anion in aortic segments from control and ovariectomized rats. Arterial sections were labelled with the oxidative dihydroethidium, which fluoresces red when oxidized to EtBr by superoxide (see Methods). The sections shown are typical of preparations from four rats. Magnification: 200x. Quantitative analysis of fluorescence is also shown. Results (mean ± SEM) are expressed as arbitrary units. Number of animals, n=4. *p<0.05 compared with control rats.

Cross sections of aorta from control and ovariectomized rat were used to evaluate the presence of superoxide anion. After incubation with hydroethidine, the arteries from ovariectomized rats showed a markedly higher level of EtBr fluorescence than the arteries from control rats. These results indicate that the level of superoxide anion was enhanced by ovariectomy (Figure *1*).

Similar results were obtained by measuring the chemiluminescence emitted by lucigenin (control, 63.2 ± 7.1 U/mg/min, n=4; ovariectomized, 345.3 ± 5.9 U/mg/min, n=6; *p* < 0.001).

### Superoxide dismutase activity

**Figure 2:**
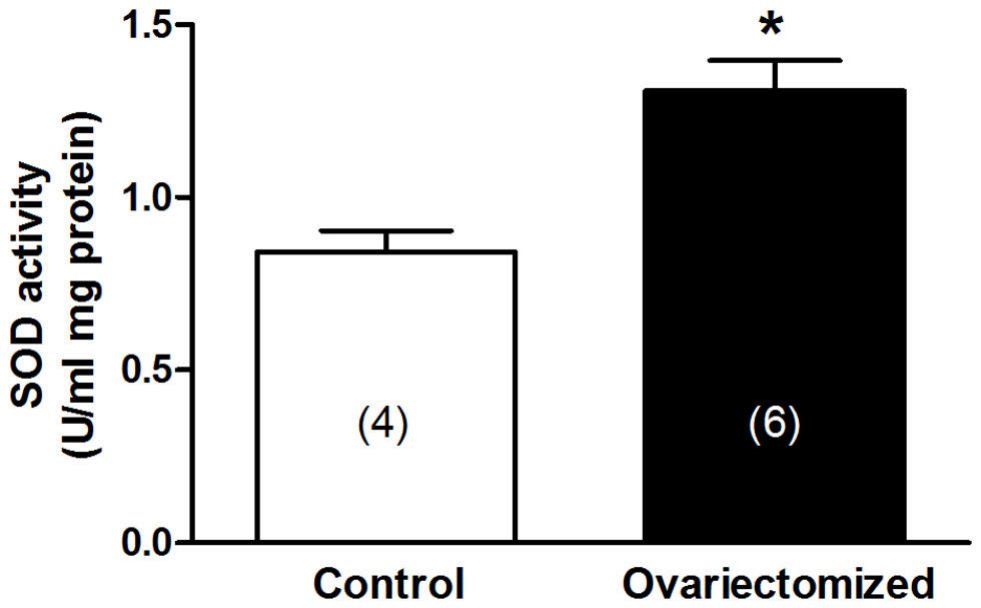
Superoxide dismutase activity in aortic segments from control and ovariectomized rats. Results (mean ± SEM) are expressed as SOD units /ml mg protein. Number of animals is indicated in parenthesis. *p<0.05 compared with control rats.

The activity of endogenous SODs was greater in aortas from ovariectomized than control rats (Figure *2*), which could indicate a compensatory mechanism to that increased superoxide anion formation.

### Determination of cGMP

**Figure 3:**
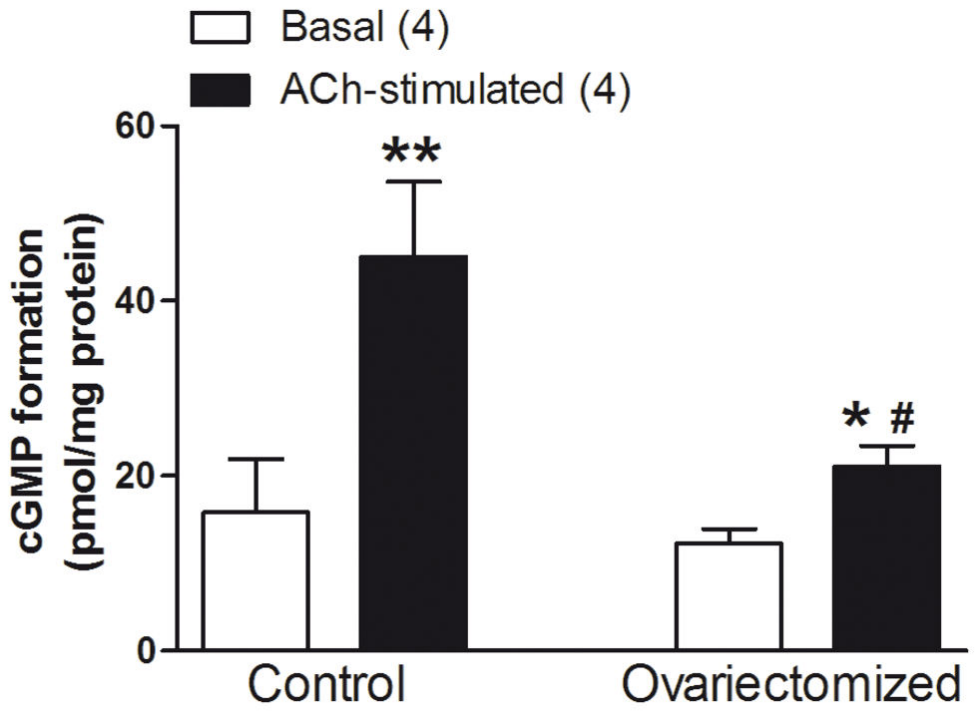
Effect of ovariectomy on the basal and ACh-stimulated cGMP formation in NA-precontracted aortic segments. Results (mean ± SEM) are expressed in pmol/mg protein. Number of animals is indicated in parenthesis. *p<0.05; **p<0.001 compared with basal condition. #*p*<0.05 compared with ACh-induced cGMP release in control rats.

Basal cGMP formation was similar in arteries from control or ovariectomized rats. ACh-induced cGMP formation was greater in aortas from control rats than in those of ovariectomized rats (Figure *3*). Therefore, we analysed the event downstream to cGMP formation, PKG activity.

### Activity of PKG

The activity of PKG was assessed in homogenates from frozen aortas. The assay showed that PKG activity was greater in aortas from ovariectomized than in those of control rats (control, 2.33 ± 0.33 A.U. /mg protein; ovariectomized, 4.75 ± 0.5 A.U. /mg protein; n = 5; *p* < 0.05). These results are inversely correlated to those of cGMP formation, indicating the existence of a cGMP-independent mechanism to activate PKG.

### Vascular reactivity

The exposure of arteries to 75 mmol/L KCl induced a contractile response that was similar in aortas from control and ovariectomized rats (control, 1345 ± 59 mg; ovariectomized, 1533 ± 123 mg; n = 15-23; *p* > 0.05). Likewise, ovariectomy did not modify the contractile response induced by 0.1 µM NA (control: 910 ± 36 mg; ovariectomized 1005 ± 48 mg; n= 15-23; *p* > 0.05).

**Figure 4:**
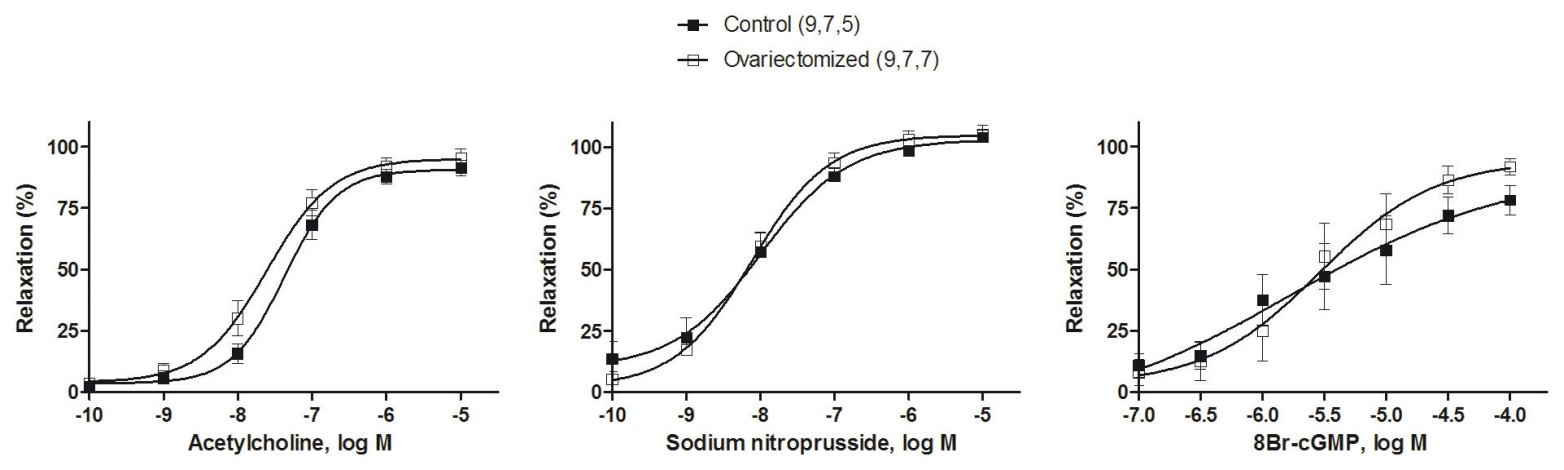
Effect of ovariectomy on the concentration-response curves to acetylcholine, sodium nitroprusside and 8Br-cGMP in rat aortic segments. Results (mean ± SEM) are expressed as percentage of inhibition of contraction induced by 0.1 µmol/L NA. Number of animals is indicated in parenthesis.

In aortic segments precontracted with NA (0.1 µmol/L), ACh (0.1 nmol/L -10 µmol/L), SNP (0.1 nmol/L -10 µmol/L) or 8Br-cGMP (0.1 µmol/L 0.1 mmol/L) induced a concentration-dependent relaxation that was similar in arteries from control or ovariectomized rats (ANOVA, *P* > 0.05; Figure *4*).

**Figure 5:**
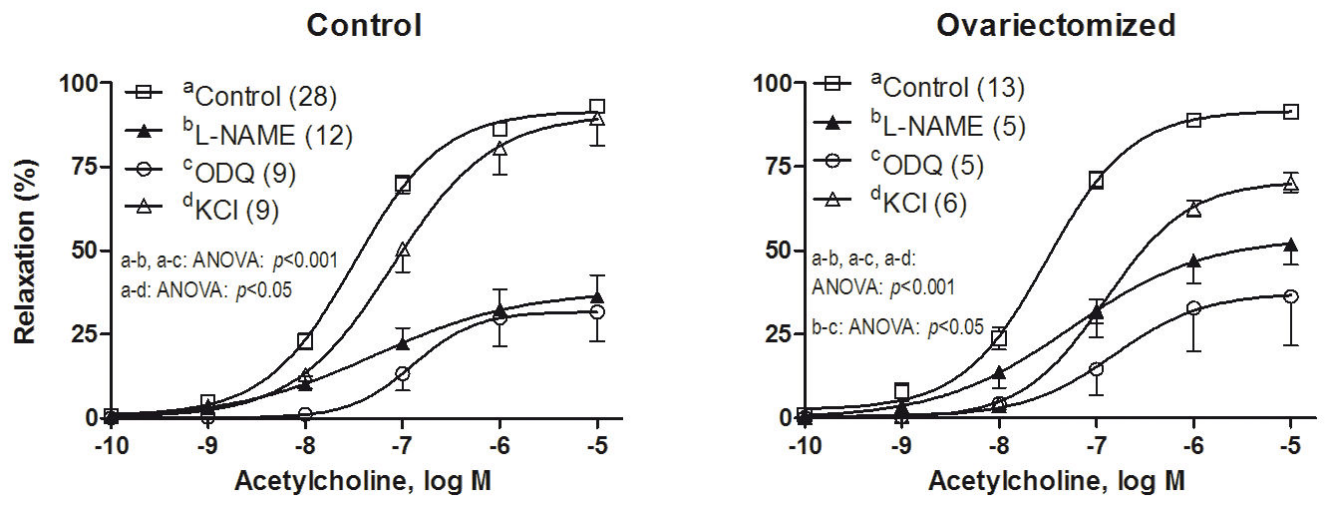
Ovariectomy modulates the participation of different factors in the acetylcholine-induced response. Effect of L-NAME (0.1 mmol/L) or ODQ (10 µmol/L) on the concentration-response curves to acetylcholine in the NA-precontracted aortic segments from control and ovariectomized rats. The effect of precontracting vessels with KCl (30 mmol/L) is also represented. Results (mean ± SEM) are expressed as percentage of inhibition of contraction induced by 0.1 µM NA or 30 mmol/L KCl. Number of animals is indicated in parenthesis.

To investigate the contribution of NO or cGMP on the vasodilator response induced by ACh (0.1 nmol/L -10 µmol/L), the effect of the inhibitors of NO synthase or guanylate cyclase, L-NAME (0.1 mmol/L) or ODQ (10 µmol/L), respectively, was examined. Preincubation with L-NAME or ODQ significantly decreased the relaxation by ACh in vessels from control and ovariectomized rats compared with the respective untreated arteries (Figure *5*; Table *1*). In the presence of L-NAME, the relaxation to ACh was significantly greater in arteries from ovariectomized than in those from control rats, while ODQ decreased the ACh-induced response in similar extent in both groups of rats (Figure *5*). These results are in agreement with the greater dAUC value observed after incubation with L-NAME in arteries from control group compared with the ovariectomized group (control, 69.2 ± 7.3%; ovariectomized, 44.4 ± 9.1%; *p* < 0.05), and similar dAUC after incubation with ODQ in both groups (control, 74.2 ± 7.5%, ovariectomized, 69.7 ± 9.7%; *p* > 0.05). An additional observation from these results is that L-NAME or ODQ evoked a similar decrease in the ACh-induced response in aortas from control rats, while in aortas from ovariectomized rats ODQ caused a greater inhibition of the ACh-induced relaxation than L-NAME did.

**Table 1:** Effect of L-NAME, ODQ and precontraction with KCl in the maximum response (E_max_) and log EC_50_ to the acetylcholine-induced response in aortas from control and ovariectomized rats.

	**Control**		**Ovariectomized**	
	**E_max_**	**log EC_50_**	**E_max_**	**log EC_50_**
**Control**	92.9 ± 1.5	-7.49 ± 0.06	91.87 ± 2.1	-7.47 ± 0.04
**L-NAME (0.1 mM**)	44.2 ± 5.1*	-7.24 ± 0.13	54.26 ± 5.5*	-7.22 ± 0.25
**ODQ (10 µM**)	31.5 ± 8.6*	-6.89 ± 0.08*	37.23 ± 5.2*	-6.73 ± 0.21>*
**KCl (30 mM**)	91.3 ± 8.8	-7.09 ± 0.13*	70.45 ± 2.8*+	- 6.92 ± 0.08*

^*^ *p* < 0.05 *vs* control condition

^+^ *p* < 0.05 *vs* control rats

Precontraction with KCl (30 mmol/L), that blocks the membrane hyperpolarization, decreased ACh-induced response more in aortas from ovariectomized rats than in those of control rats (Figure *5*; Table *1*).

**Figure 6:**
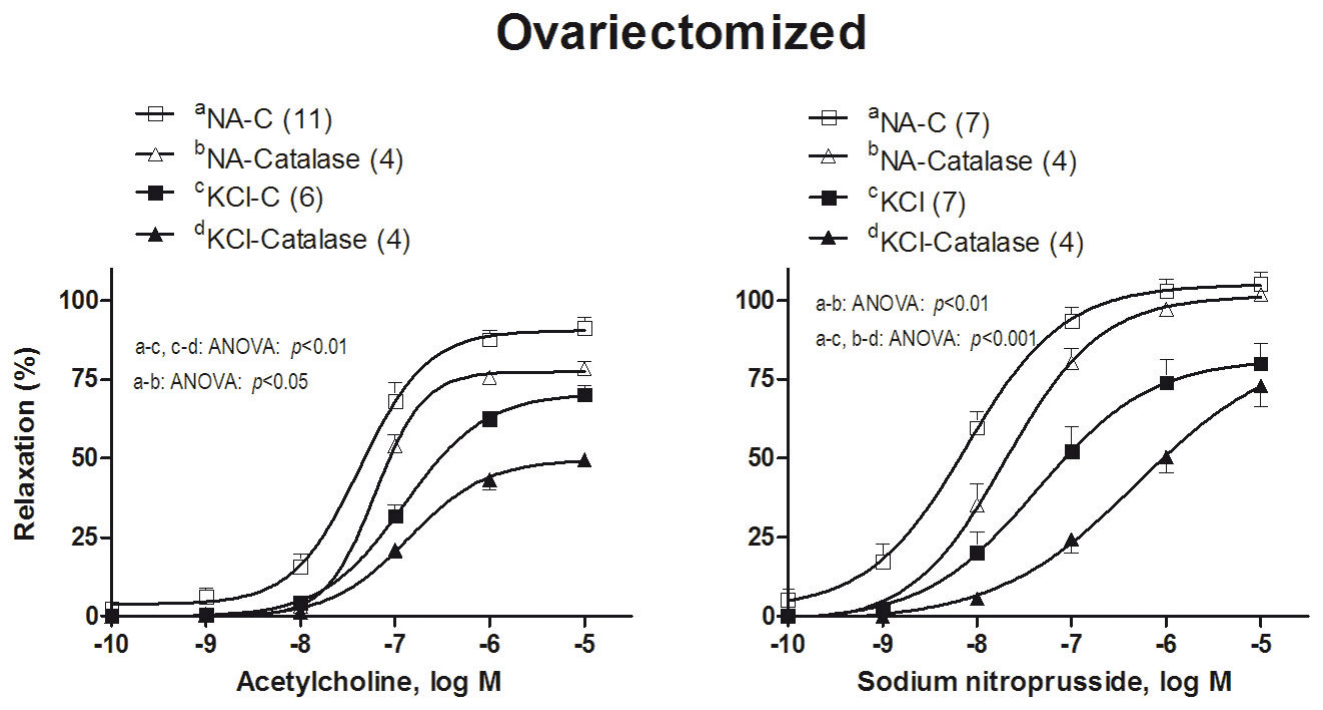
Involvement of hydrogen peroxide in the vasodilator responses to acetylcholine and sodium nitroprusside. Effect of catalase (1000 U/ml) on the concentration-response curves to acetylcholine and sodium nitroprusside in NA- or KCl-precontracted aortic segments from ovariectomized rats. Results (mean ± SEM) are expressed as percentage of inhibition of contraction induced by 0.1 µmol/L NA or 30 mmol/L KCl. Number of animals is indicated in parenthesis.

In NA-precontracted arteries, preincubation with catalase (1000 U/ml, that decompose hydrogen peroxide) did not modify the ACh-induced relaxation in aortas from control rats (data not shown), but it decreased the response in arteries from ovariectomized rats (Figure *6*); in KCl-precontracted aortas from ovariectomized rats, catalase caused a greater decrease in the ACh-induced relaxation than that which was produced in NA-precontracted vessels (Figure *6*; Table *1*)*.*

In KCl-precontracted arteries, the relaxation induced by SNP was decreased in similar extent in aortas from both control (data not shown) or ovariectomized rats. In NA-precontracted arteries, the incubation with catalase diminished the SNP-relaxation only in aorta from ovariectomized rats (Figure *6*), and catalase caused a greater decrease in the SNP-induced relaxation in KCl-precontracted arteries than in NA-precontracted arteries (Table *2*).

**Table 2:** Effect of catalase (1000U/ml) in the E_max_ and log EC_50_ to the relaxation induced by Acetylcholine and Sodium Nitroprusside in NA- or KCl-precontracted aortic segmens from ovariectomized rats.

	**Acetylcholine**		**Sodium Nitroprusside**	
	**E_max_**	**log EC_50_**	**E_max_**	**log EC_50_**
**C-NA**	91.87 ± 2.1	7.47 ± 0.04	104.8 ± 4.07	-8.12 ± 0.08
**Catalase-NA**	77.6 ± 2.07*	-7.20 ± 0.04	101.4 ± 0.8	-7.68 ± 0.13
**C-KCl**	70.45 ± 2.8*	-6.92 ± 0.08*	81.31 ± 6.36*	- 7.33 ± 0.16*
**Catalase-KCl**	49.88 ±1.8*+	-6.8 ± 0.11*	85.76 ±7.0*	-6.30 ± 0.06*+

* *p* < 0.05 *vs* NA-precontracted arteries without catalase

+ *p* < 0.05 *vs* KCl-precontracted arteries without catalase

## Discussion

In a previous work, we reported that the loss of ovarian function did not alter the ACh-induced response in rat aorta, despite the overproduction of vasoconstrictor prostanoids which upregulated endothelial NO synthase activity [19]. We also suggested that complementary cell signalling pathways -such as the NO-cGMP-PKG- could be working simultaneously to maintain the vasodilator function. Now, in the present work we provide information that reinforces our hypothesis. We observed that 5-months after ovariectomy the metabolism of NO was increased, while cGMP formation was decreased. However, endothelial NO and vasorelaxant factors, other than NO, participate in the maintained ACh-induced response by hyperpolarizing cell membrane and through hydrogen peroxide involvement. This is a novel finding, since most studies have described the importance of hyperpolarizing mechanisms mainly in resistance vessels instead of conductance. In addition, most studies analyse vascular effects of specific sex hormones while the present work provides integrative description of the alterations in signalling pathways that take place during prolonged periods after ovariectomy.

Vascular function of endothelial NO depends on its bioavailability, which is a balance between NO production and degradation. Reactive oxygen species are involved in metabolizing NO [25,26], and among them, superoxide anion plays a crucial role since it is source of many other reactive nitrogen intermediates [27]. Since estrogens have been reported to decrease oxidative stress levels [28,29], we studied the effect of ovariectomy on the *in situ* detection of superoxide anion. Experiments were undertaken by using hydroethidine fluorescence, as previously reported [23]. We observed that the fluorescence emitted by hydroethidine probe was increased in aortas from ovariectomized rats compared to those from control rats, indicating greater levels of superoxide by ovariectomy as previously reported [30]. This finding was further reinforced by using the lucigenin chemiluminescence measurement. As commented above, the level of oxygen species detected *in situ* is the result of both the production and removal of superoxide anion. Since the elimination of superoxide anion within vessel walls is performed by superoxide dismutases (SODs) that transform superoxide anion to hydrogen peroxide [31], we analyzed the activity of endogenous SODs. We observed an increased SODs activity in aortas from ovariectomized rats compared to those from control rats. This result, apparently differs from those previously published in which a decrease in the expression [30,32] and/or activity of SODs [30] has been reported. However, it is important to note that the animal model used in those studies is far different from ours, since the ovariectomy was maintained for 4-8 weeks, unlike our model in which it was maintained for 5 months, indicating the importance of the maintenance period of ovariectomy for the ovariectomy-induced vascular effects [33]. This result indicates that the increase in the activity of SODs could be a compensatory mechanism in an attempt to eliminate the elevated superoxide anion levels observed in ovariectomy, similarly to what occurs in different physiopathological conditions as hypertension [34], atherosclerosis [35], orchidectomy [36] and/or aging [37].

The elevated levels of superoxide anions metabolize the released endothelial NO. However, we did not observe any modification on NO release, at least in part, because the positive regulation that prostanoids exert on eNOS activity [19]. Therefore, we analysed the possible effect of ovariectomy in the participation of NO-dependent vasodilation in the ACh-induced response. We observed that NOS inhibition with L-NAME significantly inhibited the ACh-induced relaxation in vessels from control and ovariectomized rats, but the inhibitory effect of L-NAME was lesser in aortas from ovariectomized rats than those from control rats. As ACh-induced NO release was not modified by ovariectomy [19], this result could indicate a lesser sensitivity to NO in aortas from ovariectomized than in those from control rats, and/or the participation of relaxant factors/mechanisms other than NO in the ACh-induced response. Since the vasodilator response to the NO donor SNP was similar in arteries from control or ovariectomized rats, alterations on the sensitivity of smooth muscle cells to endothelial NO were ruled out. Based on this result, we analysed the activation of guanylate cyclase by measuring the basal and ACh-stimulated cGMP formation in arteries from control and ovariectomized rats. Our results showed that the ACh-stimulated cGMP formation was decreased in arteries from ovariectomized rats, in agreement to results previously reported [38,39]. Additionally, guanylate cyclase activity has been shown to be negatively regulated by several oxygen reactive species [40], which is in agreement with our results. Despite the decreased NO induced-cGMP formation in aortas from ovariectomized rats, the cGMP participation in the ACh-induced response was similar in aortas from ovariectomized or control rats, since the guanylate cyclase inhibitor ODQ decreased to a similar extent the ACh-induced relaxation in both groups. In addition, the cGMP analogue 8Br-cGMP induced similar relaxant response in aortas from both groups, which ruled out alterations of the vasodilator effect of cGMP, and suggest that other vasodilator mechanisms could be working to maintain the Ach-induced response in aortas from ovariectomized rats.

At this point of the discussion, it is interesting to remark the differences obtained between the two groups of animals when the synthesis of NO or cGMP is blocked. Thus, in aorta from control rats both L-NAME and ODQ induced similar degree of blockade of ACh-induced relaxation, which indicates that vascular effect of NO seems to be mediated through cGMP formation. In contrast, in aorta from ovariectomized rats the blockade of cGMP synthesis with ODQ decreased ACh-induced relaxation in greater extent than that induced by inhibiting NO synthesis, which would indicate the existence of vasodilators factors, other than NO, acting in a cGMP-independent way.

It is known that the redox environment can modify the activity of different enzymes that participate in the homeostasis of vascular tissues [41] and that sex hormones are able to modulate that effect. For example, superoxide anions produced in aortas from male rats exerts different effects depending on the presence or absence of male sex hormones. Thus, in aorta from control male rats superoxide anion metabolizes the ACh-induced NO, while in aorta from orchidectomized rats superoxide anion induces relaxation by activating calcium-dependent potassium channels [12]. Similar results were observed in mesenteric artery from orchidectomized rats in which products generated from NO metabolism, such as peroxynitrite and hydrogen peroxide, are able to induce relaxation [23]. With this background in mind and since NO, and several reactive oxygen species commented above, are able to induce relaxation through hyperpolarizing mechanisms, we analyzed the vasodilator effect of ACh in aorta precontracted with 30 mmol/L KCl, that blocks hyperpolarization by decreasing the plasma membrane potassium gradient [42]. Our results show that, under these conditions, the ACh-induced relaxation was decreased in arteries from both groups, but to a greater extent in aortas from ovariectomized rats than in those from of control rats. These results indicate the existence of a greater participation of hyperpolarizing mechanisms in aortas from the former rats. In addition, in KCl-precontracted arteries the relaxation induced by SNP was reduced to a similar extent in aortas from control or ovariectomized rats, indicating that NO hyperpolarizes equally the vascular wall in arteries from both groups of rats. Taking the results together, the differences observed in ACh-induced relaxation are probably due to vasodilator action of factors other than NO.

As has been discussed earlier, our results also show that aortas from ovariectomized rats produce more superoxide anion than those from control rats, and that SOD activity is also increased in arteries from ovariectomized rats, therefore an increased formation of hydrogen peroxide would be expected. However, enzymes other than SODs cannot be ruled out, since production of hydrogen peroxide from NADPH-oxidase has been reported [43]. Since the vasodilator effect of hydrogen peroxide has been reported [13,44], we investigated its possible role on the ACh-induced relaxation by using catalase, which decomposes hydrogen peroxide. Preincubation with catalase did not modify the ACh- or SNP-induced relaxation in NA-precontracted aorta from control rats, ruling out the functional role of endogenous hydrogen peroxide in aorta from these rats. However, in aorta from ovariectomized rats, catalase diminished the vasodilatory action of ACh and SNP, indicating the participation of hydrogen peroxide in producing relaxation. Since hydrogen peroxide has been reported to act as an EDHF [2,10,13], we analyzed, in aorta from ovariectomized rats, the effect of catalase also in KCl precontracted arteries. We observed that both ACh- and SNP-induced relaxation were reduced even further, which indicates the involvement of vasodilator mechanisms induced by hydrogen peroxide in addition to hyperpolarization. In this regard, it has been reported that hydrogen peroxide is able to activate PKG, in a cGMP-independent way, by forming the disulfide bond of this kinase [41]. Although sex difference in PKG-mediated relaxation has been described [45], the effect of endogenous female sex hormones on PKG activity in vascular tissues has not been investigated. In the present study, the analysis of PKG activity revealed an increase in aortas from ovariectomized rats, which is in line with PKG down regulation by female sex hormones observed in myometrial tissues [46]. The increased PKG activity in aortas from ovariectomized rats is in line with the hydrogen peroxide-induced PKG activation reported by Burgoyne et al. [41], and would explain the maintained response to Ach despite the decreased production of cGMP in aortas from ovariectomized rats. These results, would also explain the fact that the decrease in the ACh-induced response in the presence of ODQ does not change between control and ovariectomized rats, and support the suggestion that PKG could act as a redox sensor, in such a way that different oxidants compounds can activate the enzyme in a cGMP-independent way, participating in the maintenance of vascular homeostasis.

**Figure 7:**
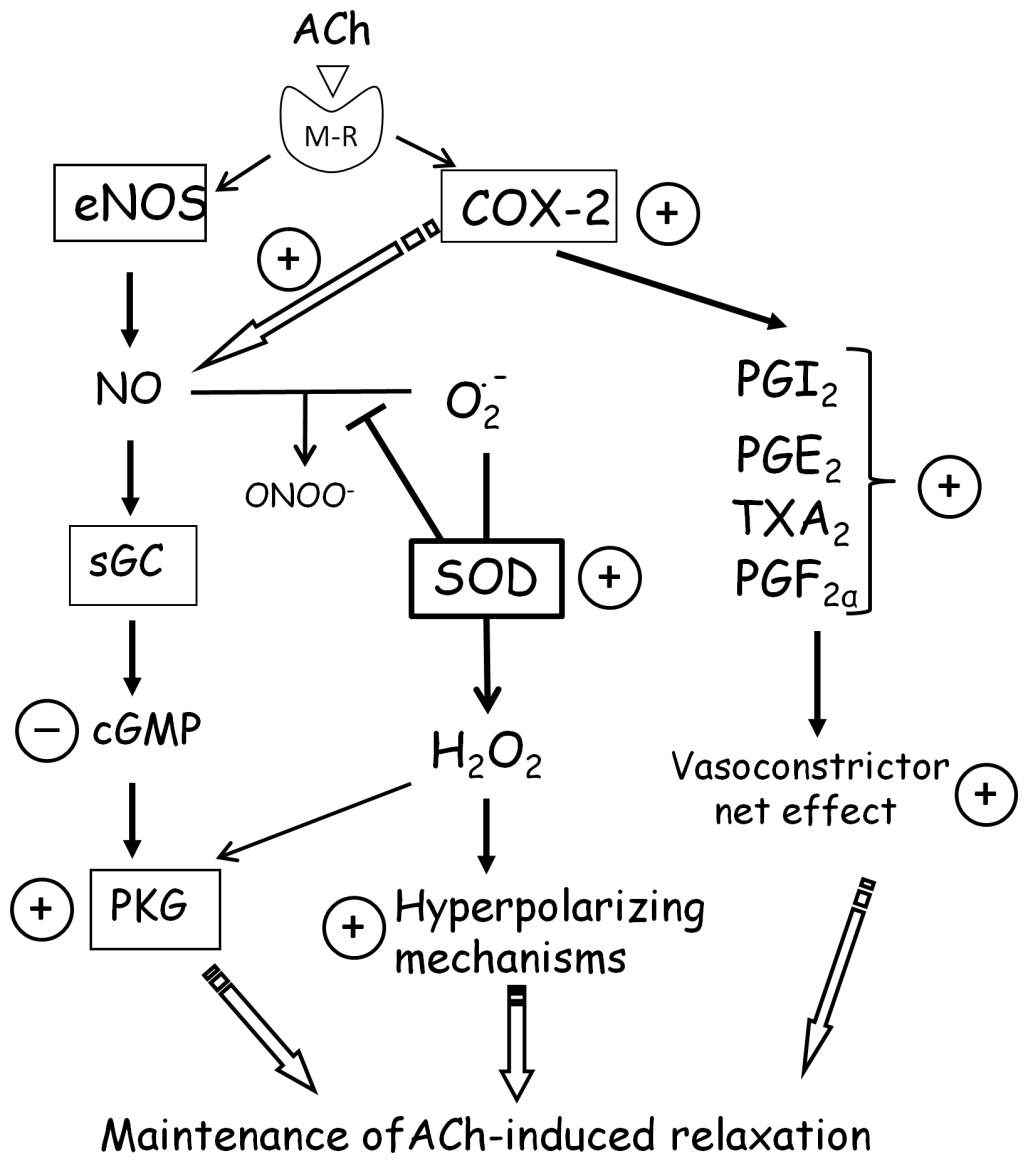
Scheme showing the involvement of the NO-cGMP-PKG and COX-2 pathways in the ACh-induced relaxation when ovarian function is lost. The encircled +/- symbols indicate the effect of ovariectomy. ACh, after binding to the muscarinic receptor (M-R), induces the release of NO and activates soluble guanylate cyclase (sGC) to produce cGMP, which is reduced by ovariectomy. However, ovariectomy increased the activity of cGMP-dependent protein kinase (PKG) that could account for the maintenance of ACh-induced vasodilation. Ovariectomy also increases the activity of superoxide dismutase (SOD) in an attempt to counterbalance the increased formation of superoxide anion (O_2_^.-^), leading to increase the participation of hyperpolarization induced by hydrogen peroxide (H_2_O_2_) and, that in turn, can activate PKG [41]. These mechanisms could counterbalance the predominance of vasoconstrictor prostanoids derived from COX-2 [19] and account for the maintenance of the ACh-induced relaxation in aortas from ovariectomized rats.

In summary, our data demonstrate that ovariectomy increases the production of superoxide anions and decreases the ACh-induced cGMP formation, which could diminish the ACh-induced relaxation in rat aorta. However, in contrast to that expected, the ACh-induced relaxation is maintained in aortas from ovariectomized rats by the existence of hyperpolarizing mechanisms in which hydrogen peroxide is involved. The greater contribution of hydrogen peroxide to the ACh-induced relaxation is probably due to the increased activity of SOD, in an attempt to compensate for the increased superoxide anion formation. In addition, the activity of PKG is increased in aorta from ovariectomized rats, which could represent a redundant mechanism to ensure vasodilator function in the aorta of these rats (see Figure 7).

These results complement those previously reported [19] and provide new insights into the mechanisms by which vasoactive factors control the vasodilator response when ovarian function is lost.
